# Association between drinking water hardness and incidence of hospitalization for childhood fracture: an ecological study of England

**DOI:** 10.1093/jbmrpl/ziaf189

**Published:** 2025-12-06

**Authors:** Clementine Kerwin, Emma M Clark, Andrew Judge, Samuel Hawley

**Affiliations:** Musculoskeletal Research Unit, Translational Health Sciences, Bristol Medical School, University of Bristol, Learning & Research Building, Level 1, Southmead Hospital, Bristol BS10 5NB, United Kingdom; Musculoskeletal Research Unit, Translational Health Sciences, Bristol Medical School, University of Bristol, Learning & Research Building, Level 1, Southmead Hospital, Bristol BS10 5NB, United Kingdom; Musculoskeletal Research Unit, Translational Health Sciences, Bristol Medical School, University of Bristol, Learning & Research Building, Level 1, Southmead Hospital, Bristol BS10 5NB, United Kingdom; Center for Statistics in Medicine, Nuffield Department of Orthopedics, Rheumatology and Musculoskeletal Sciences, Botnar Research Centre, Oxford OX3 7LD, United Kingdom; Musculoskeletal Research Unit, Translational Health Sciences, Bristol Medical School, University of Bristol, Learning & Research Building, Level 1, Southmead Hospital, Bristol BS10 5NB, United Kingdom

**Keywords:** water hardness, bone, fracture, pediatric, calcium, magnesium, mineral content, nutrition, environment, epidemiology

## Abstract

Bone fracture during childhood is a common injury, with rates in early adolescence equivalent to that of older age. Nutrition is profoundly important for healthy skeletal development, although data are scarce on the extent to which bone-forming minerals in drinking water might contribute to musculoskeletal health. The aim of this study was to describe the association between the hardness of local domestic drinking water across England with rates of hospitalization for childhood fracture, while adjusting for sociodemographic characteristics. Total water hardness (CaCO_3_) and calcium and magnesium concentrations were obtained de novo from water companies. Counts of hospitalizations for fracture in patients aged under 18 yr were extracted from the Hospital Episode Statistics database (April 1, 2012, to March 31, 2020). Sex-specific Poisson regression was used to describe the association between water hardness and rates of fracture hospitalizations aggregated at the neighborhood level (lower-level super output areas [LSOAs]), adjusted for age, deprivation, rurality and latitude. We included 29 776 LSOAs and identified 298 929 hospital admissions for fracture, at an estimated rate of 3.50/1000 person-years. Water hardness was associated with a significant reduction in fracture admissions: covariate-adjusted incidence rate ratio of 0.87 (95% CI, 0.86-0.89) and 0.84 (95% CI, 0.82-0.86) comparing very hard to soft water areas, for boys and girls, respectively. The reduction was consistent across commonly fractured skeletal sites and for secondary fractures within the same child. Concentrations of either calcium or magnesium were independently associated in a dose–response manner with fewer hospital admissions for fracture. Future research is needed to confirm and further elucidate these findings, although they are suggestive that achieving adequate dietary intake of these bone-forming minerals may be especially important for children in areas with soft drinking water. We conclude that hospital admissions for childhood fracture are approximately 10%-15% lower in hard water areas of England.

## Introduction

Approximately one-third of people experience a bone fracture during childhood, which accounts for 25% of all childhood accidents and injuries.^1^ Rates of childhood fracture peak around puberty, where they reach similar levels to older age.^1^ These can lead to decreased physical activity levels, subsequent loss of BMD^2^^,^^3^ and a significant social impact leading to school absence and affecting social interaction with peers.^4^ A childhood fracture may be a strong independent risk factor for subsequent fracture in adulthood, although evidence for this is inconsistent.^5–7^

Previously described risk factors for childhood fracture include male sex, age (incidence peaking in early–mid-teenage years), heightened activity levels, exposure to injury, obesity, and low BMD.^7–10^ Ethnic and socioeconomic variations have been observed,^11^ while rates over time are generally reported to be either stable or increasing.^8^^,^^12^^,^^13^

Nutrition is a key factor in healthy skeletal development,^14^^,^^15^ with bone-forming minerals including calcium and magnesium having been widely studied, although with inconsistent findings.^16^ Several studies have reported an inverse association between dietary intake of calcium and childhood fracture risk.^17–19^ Similarly, 2 small studies of children have suggested that low concentrations of calcium in drinking water may be associated with increased fracture risk and lower BMD.^20^^,^^21^ The World Health Organization (WHO) estimated that between 5% and 20% of dietary intake of calcium and magnesium comes from drinking water,^22^ and reported that low mineral content could adversely impact bone mineral balance, but that “detailed studies are not available”.^23^

In terms of the United Kingdom, public drinking water is sourced from different regional geologies, giving rise to significant naturally occurring variation in water hardness—principally the concentration of calcium and magnesium.^24^^,^^25^ That is, for individuals who live in areas with a hard water supply, their daily calcium and magnesium intakes are likely to be substantially higher than those living in soft water areas.^24^ This presents an excellent research opportunity to investigate the possible contribution of the long-term intake of mineral-rich drinking water on healthy skeletal development.

The primary aim was therefore to describe the association between total drinking water hardness at a neighborhood level with corresponding rates of hospitalization for pediatric fracture across England. Secondary objectives included investigating individual concentrations of calcium and magnesium, and investigating childhood hospitalization for specific fracture types, including secondary fractures.

## Materials and Methods

### Study design and participants

The chosen study design was ecological, with the unit of observation being at the level of lower-layer super output area (LSOA) as defined for the year 2011.^26^ Each LSOA comprises between 400 and 1200 households, and there were 32 844 LSOAs in England during the study period. This unit of analysis was chosen as it was the most granular level we could use for deriving both exposure and outcome estimates. The population studied included the number of boys and girls (aged <18 yr) within each LSOA for the years 2012-2019, as recorded by midyear estimates from the Office for National Statistics (ONS). These years were used given that they corresponded to years for which we had data access to hospitalizations for fracture.

### Exposure data

The main exposure was the average total hardness of local drinking water for the year 2012. Water hardness is caused by dissolved calcium and magnesium but is expressed as the concentration of calcium carbonate (CaCO_3_ mg/l).^25^^,^^27^ Each water company in England divides the geographical area under their control into water supply zones (WSZs), such that the composition of drinking water is the same for all consumers within a WSZ.^28^ We wrote to all major water companies or subsidiaries administering a public water supply in England (Table 1), requesting values of total water hardness in each of their WSZs for the year 2012. We also requested individual concentrations of calcium (mg/l) and magnesium (mg/l), and a postcode-based lookup for the geographical area within each WSZ, if available. These data were used in conjunction with postcode lookups provided by the ONS^26^ to derive average exposure estimates at the level of LSOA (for comparability with hospital data). Where one postcode was supplied by multiple WSZs, an average of the hardness values from each WSZ was taken. Slightly lower granularity or concentrations for 2013 (rather than 2012) had to be used for some LSOAs given the availability of exposure data provided by water companies (Table 1). Any LSOAs for which we were unable to estimate water hardness were excluded from subsequent analyses. Hardness (CaCO_3_) categories in main analyses were derived as used by the Drinking Water Inspectorate for England and Wales^25^: soft (0-99 mg/l), slightly hard (100-149 mg/l), moderately hard (150-199 mg/l), hard (200-299 mg/l), and very hard (≥300 mg/l).

### Outcome data

Fracture admissions were extracted from the Hospital Episode Statistics (HES) database using a previously published list of International Classification of Diseases, Tenth Revision (ICD-10), codes for all types of fracture, sourced from the London School of Hygiene and Tropical Medicine Data Compass,^29^ modified to exclude tooth fractures (Table 2). All publicly funded hospital admissions in England are included in the HES database, and we extracted admissions for fracture between April 1, 2012, and March 31, 2020. We grouped these as follows: forearm, upper-arm, leg, skull/face, hand/foot, and other (including pelvis, rib, spine/neck, thorax, and other). In main analyses, where hospital admissions contained multiple fracture events then only the first coded fracture was considered, although in secondary analyses each skeletal site was counted separately (including across admissions with multiple fractures). Suspected duplicate coding of clinical events were excluded, defined as entries for subsequent admissions for fractures at the same skeletal site as an index event if occurring within a 6-mo time frame.^30^ Patient residence is collected at hospital admission using a postcode, which is then captured in HES data at the level of LSOA. Counts of hospital admissions due to fracture during the study period were aggregated for each LSOA included in the study—overall and stratified by sex. Counts of secondary/subsequent fractures within the same individual were likewise aggregated. In main analyses, fractures were included irrespective of mechanism, with a sensitivity analysis conducted excluding admissions related to road traffic accidents.

### Statistical analysis and data visualization

Overall and sex-stratified incidence rates (per 1000 person-years) of hospital admissions for childhood fracture were estimated using Poisson regression models including the log of the at-risk population as an offset and robust Huber-White SEs.^31^^,^^32^ Crude and multivariable models were used to describe the association between aggregated fracture counts (by LSOA) and water hardness categories, separately for boys and girls. Multivariable models were adjusted for the age distribution of the at-risk population within each LSOA (aged <9 yr, 10-12 yr, and 13-17 yr) and index of multiple deprivation (IMD) quintile.^33^ They also included a north vs south identifier based on LSOA latitude in order to address possible meteorological differences, and an identifier of urban-rural classification (rural, semi-urban, and urban) linked using freely available ONS data,^34^ given that rurality has previously been found to be a risk factor for childhood fracture.^35^ In main analyses, the hardness category “Soft 0-99 mg/l” was used as a baseline reference to which other hardness categories were compared. Results are reported as incidence rate ratios (IRRs) with 95% CIs and *p*-values.

In secondary analyses, quartiles were derived for individual calcium and magnesium concentrations among the subgroup of LSOAs for which water companies were able to provide these data. Associations between these calcium and magnesium concentrations with overall fracture hospitalizations were estimated using Poisson models (with robust SEs) adjusted as per the main models plus mutual adjustment for each other. In further secondary analyses, a binary exposure of <200 mg/l vs ≥200 mg/l CaCO_3_ (reflecting the “hard” cut point used by the Drinking Water Inspectorate^25^) was used to model rates of overall fracture admissions, secondary fracture admissions, and rates for individual skeletal sites. Given the lower number of outcome events at specific skeletal sites, these models were not stratified by sex and so were further adjusted for the sex ratio of the at-risk population in each LSOA.

In sensitivity analyses, main models for total water hardness were re-run; first, following exclusion of hospital admissions containing a road traffic accident trauma code; second, using a conservative approach using the nine “Government Office” geographic regions entered as a random intercept (which were adjusted rather than stratified for sex in order to achieve model convergence); third, stratified by IMD in order to explore the consistency or otherwise of findings depending on an LSOA’s socioeconomic deprivation; and fourth, re-run including only LSOAs with water hardness estimated using full rather than partial postcode (as detailed in Table 1). Further sensitivity linear regression models were used to model the covariate-adjusted association between incidence rates of fracture outcomes and CaCO_3_ as a continuous variable, although this sensitivity analysis excluded the area covered by South West Water, given that we were unable to link exposure data on the continuous scale for that area.

Stata/MP 18.0 was used for analyses and to create maps, using Shapefiles downloaded from the ONS website. In exploratory mapping, LSOA-level visualization was overly granular, so we opted to use the more clinically meaningful geographical area of National Health Service (NHS) Clinical Commissioning Groups (CCGs) for these maps, which were operative in England for the majority of the study period. All other statistical modeling was carried out at the LSOA level. For mapping purposes only, we also combined the “slightly” and “moderately” hard water categories for ease of visualization and reported average coverage (percentage of included LSOAs) per CCG. Stata packages used were spmap, geo2xy, palettes, colrspace, and schemepack.

## Results

### Total hardness

Of the 32 844 LSOAs across England during the study period, 29 776 (~90%) were included in main analyses (Table 1), representing a total of 85.3 million at-risk person-years across the study period. The remaining ~10% of LSOAs were excluded given that we could not estimate water hardness for these areas. The average annual at-risk population for each included LSOA was 358 (SD, 127), which was mainly composed of preadolescent children (Table 1).

**Table 1:** Characteristics of underlying denominator population within included study areas (LSOAs) during the study period.

	Soft, *n* = 7150 LSOAs		Slightly hard, *n* = 2739 LSOAs		Moderately hard, n = 2465 LSOAs		Hard, *n* = 12 971 LSOAs		Very hard, *n* = 4451 LSOAs		Overall, *n* = 29 776 LSOAs		*p*-Value
	Person-years	Row %	Person-years	Row %	Person-years	Row %	Person-years	Row %	Person-years	Row %	Person-years	Column %	
Annual average population at-risk per LSOA (SD)	352 (135)		346 (127)		338 (118)		366 (125)		364 (123)		358 (127)		<.001
Total denominator at-risk (million)	20.1	24	7.6	9	6.7	8	38.0	45	12.9	15	85.3	100	<.001
By sex													
Boys	10.3	24	3.9	9	3.4	8	19.4	44	6.6	15	43.7	51	
Girls	9.8	24	3.7	9	3.3	8	18.6	44	6.3	15	41.6	49	<.001
By age													
Aged ≤9 yr	11.5	23	4.3	9	3.8	8	22.1	45	7.4	15	49.1	58	
Aged 10–12 yr	3.3	24	1.2	9	1.1	8	6.0	44	2.1	15	13.7	16	
Aged ≥13 yr	5.4	24	2.0	9	1.8	8	9.8	43	3.5	15	22.6	26	<.001
By deprivation													
Quintile 1 (least deprived)	2.5	15	1.4	9	1.4	9	7.7	47	3.4	21	16.3	19	
Quintile 2	2.8	18	1.3	8	1.3	8	7.3	47	2.9	18	15.7	18	
Quintile 3	3.2	20	1.3	8	1.3	8	7.5	47	2.8	18	16.1	19	
Quintile 4	4.0	23	1.3	8	1.2	7	8.5	49	2.3	14	17.3	20	
Quintile 5 (most deprived)	7.7	39	2.3	12	1.5	8	7.0	35	1.4	8	19.9	23	<.001
By rurality													
Rural	2.6	19	1.1	8	1.2	9	5.4	40	3.2	24	13.4	16	
Semi-urban	5.5	15	4.1	11	2.7	8	15.8	44	7.7	22	35.8	42	
Urban	12.1	33	2.4	7	2.7	7	16.8	46	2.1	6	36.2	42	<.001
By latitude													
South	4.4	8.7	2.7	5.3	3.1	6.1	30.0	59.2	10.6	20.9	50.7	59.4	
North	15.8	45.7	4.9	14.2	3.6	10.4	8.0	23.1	2.4	6.9	34.6	40.6	<.001

Percentages/totals may not sum exactly due to rounding.

Abbreviation: LSOA, Lower-Level Super-Output Area.

**Figure 1:**
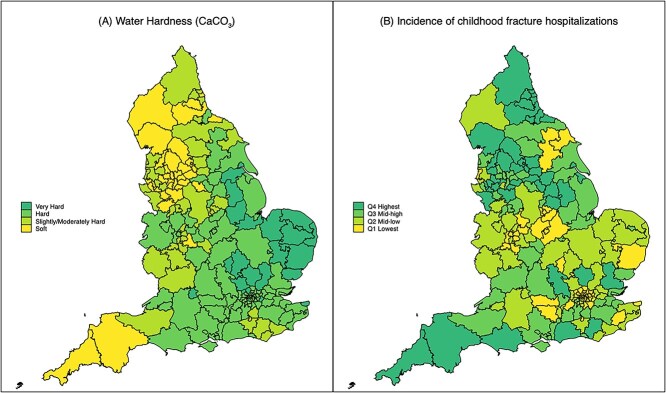
Geographical variation across England in average local drinking water hardness and crude incidence of hospitalizations for childhood fracture: by NHS Clinical Commissioning Group. Average sample coverage per Clinical Commissioning Group: 98% (IQR, 88%-100). Abbreviations: NHS, National Health Service.

Figure 1A shows the geographical distribution of average hardness at the level of CCG, showing many of the hardest water areas arise in the East of England and parts of the East Midlands and South East, whereas the softest water supplies originate from Cornwall, Devon, and the North West (although over 5% of the LSOAs estimated as having soft water were located in each of the South West, North West, North East, West Midlands and Yorkshire/Humber). Average sample coverage (i.e proportion of eligible LSOAs included) per CCG was 98% (IQR 88-100%). For the 29 041 LSOAs with water hardness values on a continuous rather than categorical scale, overall median hardness was 246.2 (IQR, 119.8-283.2) mg/l CaCO_3_, although there was a bimodal distribution with peaks around 50 mg/L and 280 mg/L.

A total of 298 929 hospital admissions for fracture were identified during the study period, of which 9873 (3.3%) were for secondary fractures. This corresponded to an estimated overall rate of 3.50 (95% CI, 3.49-3.52)/1000 person-years, which was higher in boys (4.56 [4.53-4.58]) than in girls (2.41 [2.39-2.43]), and for which there was notable geographic variation (Figure 1B). The majority of admissions were due to fractures sustained at the forearm (39%) or leg (17%).

**Figure 2:**
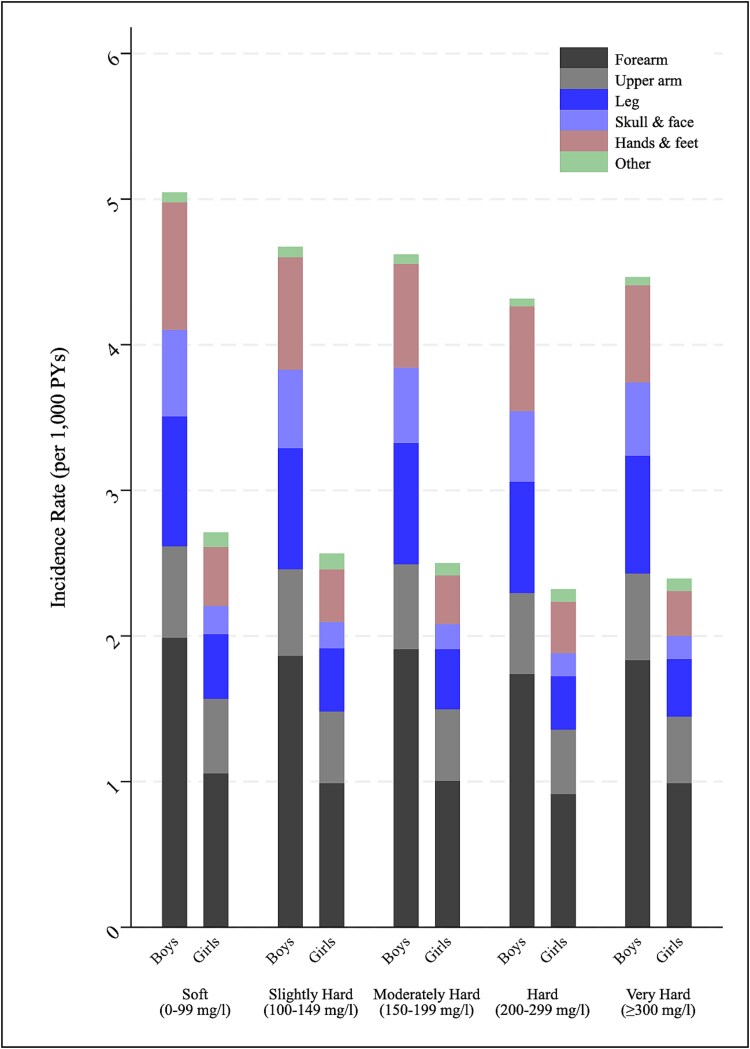
Sex-specific crude incidence of hospitalizations for childhood fracture: by hardness of local drinking water (CaCO_3_). Abbreviation: PY, person-year.

There was a significant downward trend in incidence of fracture-related hospital admissions with increasing water hardness (Figure 2, Tables 2 and 3). Compared with soft water, covariate-adjusted models showed an approximate dose–response reduction from slightly hard (IRR, 0.91 [0.89-0.93] and 0.91 [0.87-0.94] for boys and girls, respectively) to very hard water (IRR, 0.87 [0.86-0.89] and 0.84 [0.82-0.86], respectively). Findings were identical in sensitivity models excluding 33 732 hospital admissions for road traffic accidents: very hard water–adjusted IRR of 0.86 (0.85-0.88) for males and 0.84 (0.82-0.87) for females. Findings were also similar in sensitivity multilevel models irrespective of covariate adjustment, with a downward trend observed across all categories, albeit estimated reductions were slightly smaller: IRR of 0.93 (0.92-0.95) for very hard water (≥300 mg/l) compared with soft water. Associations were likewise consistent across different strata of deprivation (Table 3) and in analyses only including LSOAs with water hardness estimated using full rather than partial postcodes: very hard water adjusted IRR of 0.91 (0.88-0.93) and of 0.89 (0.87-0.91) for boys and girls, respectively.

**Table 2:** Number and incidence rate ratio for fracture hospitalizations according to total water hardness and calcium and magnesium concentrations among boys.

	Fracture admissions, *n*	Crude		Adjusted^a^	
		Incidence rate ratio	*p*-Value	Incidence rate ratio	*p*-Value
Total hardness^b^					
Soft (0-99 mg/l)	52 177	Reference		Reference	
Slightly hard (100-149 mg/l)	18 080	0.92 (0.90–0.94)	<.001	0.91 (0.89–0.93)	<.001
Moderately hard (150-199 mg/l)	15 760	0.91 (0.89–0.93)	<.001	0.91 (0.89–0.93)	<.001
Hard (200-299 mg/l)	83 457	0.85 (0.84–0.86)	<.001	0.87 (0.86–0.88)	<.001
Very hard (≥300 mg/l)	29 415	0.88 (0.86–0.89)	<.001	0.87 (0.86–0.89)	<.001
					
Calcium^b^					
Quartile 1 - lowest	43 397	Reference		Reference	
Quartile 2	38 780	0.94 (0.92–0.95)	<.001	0.98 (0.96–1.00)	.027
Quartile 3	39 108	0.90 (0.89–0.92)	<.001	0.96 (0.94–0.98)	<.001
Quartile 4 - highest	39 126	0.89 (0.87–0.90)	<.001	0.94 (0.92–0.96)	<.001
					
Magnesium^b^					
Quartile 1 - lowest	48 853	Reference		Reference	
Quartile 2	42 632	0.89 (0.88–0.91)	<.001	0.96 (0.94–0.98)	<.001
Quartile 3	43 290	0.90 (0.88–0.91)	<.001	0.94 (0.92–0.95)	<.001
Quartile 4 - highest	41 187	0.89 (0.87–0.90)	<.001	0.89 (0.87–0.90)	<.001

a Adjusted for age, deprivation (index of multiple deprivation [IMD]), latitude, and rurality. Models for calcium and magnesium were also mutually adjusted for calcium/magnesium concentration.

b Lower-layer super output areas included *n* = 29 776 for total water hardness and *n* = 21 858 for calcium and magnesium.

**Table 3:** Number and incidence rate ratio for fracture hospitalizations according to total water hardness and calcium and magnesium concentrations among girls.

	Fracture admissions, *n*	Crude		Adjusted^a^	
		Incidence rate ratio	*p*-Value	Incidence rate ratio	*p*-Value
Total hardness^b^					
Soft (0–99 mg/l)	26 181	Reference		Reference	
Slightly hard (100–149 mg/l)	9264	0.94 (0.92–0.97)	<.001	0.91 (0.87–0.94)	<.001
Moderately hard (150–199 mg/l)	8033	0.93 (0.90–0.96)	<.001	0.91 (0.89–0.94)	<.001
Hard (200–299 mg/l)	41 805	0.85 (0.83–0.86)	<.001	0.85 (0.83–0.87)	<.001
Very hard (≥300 mg/l)	14 757	0.88 (0.86–0.90)	<.001	0.84 (0.82–0.86)	<.001
					
Calcium^b^					
Quartile 1 - lowest	21 599	Reference		Reference	
Quartile 2	19 817	0.96 (0.94–0.98)	<.001	0.99 (0.97–1.02)	.54
Quartile 3	19 802	0.92 (0.90–0.94)	<.001	0.95 (0.93–0.98)	<.001
Quartile 4 - highest	19 708	0.90 (0.88–0.92)	<.001	0.94 (0.91–0.96)	<.001
					
Magnesium^b^					
Quartile 1 - lowest	24 124	Reference		Reference	
Quartile 2	21 908	0.93 (0.91–0.95)	<.001	1.01 (0.99–1.04)	.28
Quartile 3	21 518	0.90 (0.88–0.92)	<.001	0.95 (0.93–0.98)	<.001
Quartile 4 - highest	20 968	0.91 (0.89–0.93)	<.001	0.90 (0.88–0.93)	<.001

a Adjusted for age, deprivation (index of multiple deprivation [IMD]), latitude, and rurality. Models for calcium and magnesium were also mutually adjusted for calcium/magnesium concentration.

b Lower-layer super output areas included *n* = 29 776 for total water hardness and *n* = 21 858 for calcium and magnesium.

In secondary analyses using a binary exposure of water hardness (</≥200 mg/l CaCO_3_), overall hospitalizations for fracture were 10% lower in areas with harder water: adjusted IRR, 0.90 (0.89-0.91); *p* < .001 (Table 4). A reduction of a similar magnitude was observed across all skeletal sites and in the incidence rate of secondary fracture: IRR = 0.87 (0.83-0.91) (Table 4). Modeling of hardness as a continuous variable indicated an overall fracture rate reduction of 16/100 000 person-years associated with each increase of 100 mg/l in total hardness (Table 4).

### Calcium and magnesium

Several water companies could not provide individual calcium and magnesium concentrations as requested. Among the subgroup of LSOAs in which individual concentrations of calcium and magnesium could be estimated (*n* = 21 858), the median concentration of calcium was 58.5 (IQR, 28.4-97.1) mg/l and for magnesium was 5.5 (2.7-9.0) mg/l. Covariate-adjusted rates of hospitalization for fracture showed an approximate dose–response reduction across concentration quartiles of both calcium and magnesium—suggesting reductions of approximately 5% and 10% in the highest vs lowest quartiles of calcium and magnesium, respectively (Tables 2 and 3).

### Sociodemographic factors

Associations between the incidence of hospitalization for fracture and sociodemographic adjustment factors were not the main subject of the study but are reported in Table 5. Mutually adjusted estimates suggested that rates of fracture hospitalizations were significantly (*p* < .001) lower for children living in urban compared with rural areas (IRR, 0.89 and 0.81 for boys and girls, respectively), while were elevated for boys in areas with greater deprivation (IRR, 1.13 for the most deprived quintile) but elevated for girls in areas further north (IRR, 1.04).

## Discussion

The results of this national study suggest that hard drinking water areas are associated with a lower incidence of childhood fracture, with approximately 10%-15% fewer hospital admissions for fracture as compared with areas with soft drinking water. Secondary analyses suggested that fracture admissions were lower in areas with higher drinking water concentrations of either calcium or magnesium, with reductions consistent across different skeletal sites and for secondary fractures.

Our geographical variation in water hardness (Figure 1A) is consistent with that reported by the UK Drinking Water Inspectorate,^36^ albeit the granularity in Figure 1 (at the level of CCG) is lower. Likewise, our main findings are consistent with the limited existing literature correlating mineral levels in water with pediatric fracture rates.^20^^,^^21^ A previous study in China investigating the effects of different mineral concentrations in drinking water found that children who drank water with very low concentrations of calcium and other minerals also had decreased BMC and osteoblast activity.^20^ A small study from Spain found a statistically significant relationship between low calcium levels in water supplies and increased fracture incidence.^21^ On the other hand, historical data on calcium concentrations at the level of UK counties found no significant linear association with adult hip fracture rates.^37^ Other UK population–based studies on the potential impact of hard water on varied health conditions are emerging.^38–40^

Given that calcium and magnesium in drinking water can make up a significant proportion of overall dietary intake of these bone-forming minerals,^22^^,^^24^ there is strong biological plausibility for the observed reduction in childhood fracture rate associated with long-term higher exposure to harder drinking water. The central role of calcium in healthy bone growth is well documented, with early puberty suggested as the time of greatest calcium requirement.^41^ Where intake is insufficient, the body may start to take calcium from bone, which, over time, can lead to bone weakness and increased risk of fracture.^42^ Studies of calcium intake have supported a positive role on influencing bone mineralization during childhood and adolescence,^41^ although supplement interventional studies have generally found a negligible effect on fracture risk.^43^ The effect of calcium specifically from dairy sources has shown more sustained benefits than from salts, but isolating a causal effect of calcium from benefits of growth factors and proteins (eg, in milk) is challenging.^16^ As such, debate continues regarding the extent to which calcium per se has a sustained beneficial effect in terms of optimizing bone growth and development.^14^^,^^16^^,^^44^

In terms of magnesium—the second most abundant intracellular cation—existing data are conflicting on a role in bone health, although many studies have reported that higher dietary and/or serum concentrations are associated with higher BMD and lower fracture risk.^45–47^ Higher magnesium intake has been associated with increased physical activity,^48^ which has been found to be associated with higher BMD in children (although also higher fracture risk for vigorous physical activity, most likely due to exposure to injuries).^10^ The recommended daily intake for children varies according to age.^49^ Although median concentrations in our dataset were well below recommended daily amounts, some areas had water containing more than 30 mg/l of magnesium (which is consistent with previous reports^24^), meaning that pre-teenage children consuming 1.5 l of such water per day could potentially be ingesting up to 35% of their recommended daily allowance through this means. The effects of magnesium on bone phenotype is less well studied than for calcium, and future high-quality randomized controlled trials of magnesium supplementation are likely to be needed to determine any therapeutic implications for fracture protection.^50^

The disentangling of total water hardness into separate concentrations of calcium and magnesium added additional insight into our main findings on water hardness, albeit this could only be performed in a subgroup. These secondary analyses suggested that the reductions in hospital admissions for fracture observed in areas with the highest quartile concentration of magnesium (10%-11% reduction, depending on sex), and to a slightly lesser extent calcium (6% reduction), were similar to that observed for overall water hardness (13%-16% reduction, depending on sex). While the observed dose-response association fulfills one of the Bradford Hill criteria for assessing causality, It must be noted that we were unable to account for other factors (including lifestyle, environmental, health conditions, medication use, and others) that would likely interact to some extent with intake and bioavailability of these minerals to influence bone health.^14^

A related limitation is the ecological study design, which meant that we were not able to prove a cause–effect relationship. Individual exposure was not measured at the level of individual children but rather aggregated at the neighborhood level (LSOA), which may have led to exposure misclassification. For example, some children may go to school in a different LSOA than their home and drink tap water there, or drink bottled mineral water, or drink very little water at all. This could possibly have contributed to the crude dose–response trend being somewhat attenuated in the very hard water category (Figure 2), if, for example, homes in areas with domestic water readily forming limescale in pipes/appliances were more likely to fit water-softening devices. Despite this, sample coverage was excellent and the unit of analysis was highly granular with over 29 000 observations, allowing for adjustment of age, sex, deprivation, and rurality. Our estimates were robust to the additional adjustment of a north/south identifier, which aimed to address potential for differences in sunlight exposure (and therefore vitamin D status). Nonetheless, we cannot rule out residual confounding caused by other drinking water parameters (eg, trace elements) or geographical variation in other relevant individual-level or area-level factors for which a person-level analysis may be required. We were also unable to model any temporal changes in water hardness concentrations, although this is largely determined geologically and therefore does not vary much over time.

The HES database covered all publicly funded admissions in England, although this meant we were unable to investigate Scotland or Wales, both of which contain a large proportion of the soft water in the United Kingdom. However, a large majority of all LSOAs in England were included in this study, and the exposure data contained a good variation of water hardness. It is unlikely that the LSOAs not included in the study would be systematically different than those that were included, given that exclusion was only based on several water companies being unable to provide information to convert WSZ-based data to LSOA-based (for the sake of correlating with hospital data). The very large sample representative of the whole of England up to 2020 is a considerable strength, permitting a well-powered, sex-stratified analysis of rates and investigation into admissions for individual skeletal fracture sites and secondary fractures. The observed reduction in rates of skull and digit fractures is interesting as in adults these are normally considered more the consequence of trauma rather than fragility. These associations for skull and digit fractures remained identical in sensitivity analyses excluding road traffic accidents (results not shown). Foot and hand fracture risk has previously been found to be associated with lower bone mass,^51^ and so better bone health during development could reasonably decrease the risk of contemporaneous fractures across these sites.

However, the use of HES meant that we were only able to consider fractures that led to hospital admission; primary care data were not considered. We also acknowledge the lack of validation of the code list used to identify fracture admissions, which also likely introduced some misclassification bias—for example, through omission of more minor fractures treated in outpatient or private settings. This meant that fractures managed in the community were unaccounted for, although a conservative approach means we are relatively confident that fracture counts refer to true clinical events rather than suspected diagnoses or parental over-diagnosing.^52^ It is difficult to gauge how such misclassification might impact findings, although 1 possible suggestion would be that the issue should be nondifferential according to water hardness status, in which case meaning that our estimates would be more likely to be biased towards the null. We cannot rule out that the gradient in deprivation associated with water hardness (Table 1) might have led to greater use of private healthcare (not covered in our study sample) in harder water areas, although it is reassuring that reductions in fracture rates were similar within each quintile of deprivation (Table 3) and for major fracture types (Table 4), which would almost certainly present at a public accident and emergency department. Reassuringly, our observed variations in fracture admissions by sex and region are consistent with previous literature on the epidemiology of childhood fractures as diagnosed in UK primary care, although our overall rates are understandably lower.^11^ In an exploratory post hoc analysis, we used these previously published childhood fracture incidence rates diagnosed in primary care (reported at the regional level) and explored the association between these and our water hardness data.^11^ These showed a small decrease in rates with increasing concentrations of water hardness, although we did not formally assess statistical significance given the lack of granularity and sample size (Figure 1).

Given the study design used, an ecological fallacy must be avoided in terms of inappropriately concluding that the higher fracture rates in soft water areas were definitely due to the lower mineral intake of children drinking softer water. As such, results may be more hypothesis-generating, with future research at the person-level recommended before any firm conclusions can be drawn.^53^ Future studies are also needed to further specify the mechanisms underpinning our observed associations, and investigate whether they are observed elsewhere in the world (England is not the only country to experience a geographic variation in water hardness). Identification of high-risk groups is an important step in the right direction to promote fracture prevention and bone health education. Future research could explore whether children in areas of soft water supplies might especially benefit from support to maintain healthy diets that achieve adequate calcium and magnesium intake. Our findings also suggest that this may be particularly important for those who have already sustained a first fracture—for example, through a potential role in fracture healing—although any implications on management of fracture remains a subject for further research. Given the high prevalence of childhood fracture in the general population (Tables 2 and 3), even modest reductions in risk could translate to significant numbers of fractures prevented at a population level.

## Conclusion

Hospital admissions for childhood fracture in England are approximately 10%-15% lower in areas with hard drinking water. These findings suggest that achieving adequate intake of bone-forming minerals through a healthy diet may be especially important for children in areas with soft drinking water, although future studies are needed to confirm and further elucidate the relationship.

## Supplementary Material

Supplementary_Material_ziaf189

## Data Availability

Hospital Episode Statistics (HES) data were re-used with the permission of the Health and Social Care Information Centre (“NHS Digital”). All rights reserved. HES data are available on request from NHS England’s Data Access Request Service (DARS), subject to approval and based on an appropriate user case and legal basis. Exposure data provided to the study researchers by water companies are available directly from individual water companies.
