# Supplementary material for: Association between drinking water hardness and incidence of hospitalization for childhood fracture: an ecological study of England
Source: JBMR Plus. 2025 Dec 6;10(4):ziaf189. doi: 10.1093/jbmrpl/ziaf189 (PMC12965205; doi:10.1093/jbmrpl/ziaf189)

Supplementary Materials

Supplementary Table 1: List of water companies and inclusion in the study……….. Page 1
Supplementary Table 2: List of ICD10 codes used to identify fracture admissions … Page 2
Supplementary Table 3: Adjusted incidence rate ratio for fracture hospitalizations according to total water hardness: by quintiles of deprivation…………………………………..... Page 9
Supplementary Table 4: Adjusted incidence rate ratios for fracture hospitalisation derived in secondary analyses: overall and by fracture type …………………………………… Page 10
Supplementary Table 5: Full list of crude and adjusted associations between total water hardness, case-mix factors, and overall fracture incidence in main analysis ……….…. Page 11
Supplementary Figure 1: Scatter plot of regional primary care fracture rates (previously published) by water hardness ……………………………………………………….. Page 12

Supplementary Table 1: List of water companies and inclusion in the study

**Water companies from whom exposure and/or postcode look-up information was requested**	**Data provided enabled inclusion**	**Notes**
Affinity	Yes	
Anglian Water	Yes	WSZ-LSOA linkage achieved through combination of parish and postcode
Bristol Water	Yes	Data provided for 2013 as 2012 unavailable
Bournemouth Water	No	Unable to provide in format required for analysis
Cambridge Water	Yes	Data provided for 2013 as 2012 unavailable
Essex and Suffolk Water	Yes	
Hartlepool Water	No	Unable to provide for 2012 nor postcode lookups for linkage
Northumbrian Water	Yes	WSZ-LSOA linkage achieved using outer rather than full postcode (LSOAs supplied by >1 WSZ remained as missing/excluded)
Portsmouth Water	Yes	
Sutton and East Surrey Water	Yes	
South East Water	Yes	WSZ-LSOA linkage achieved using postcode sector rather than full postcode
Southern Water	Yes	Unable to provide data on Ca or Mg for 2012
South Staffordshire Water	Yes	
Severn Trent Water	Yes	
South West Water	Yes	Provided concentrations but unable to provide look-up data. However all WSZs <100 mg/l $CaCO_3$ except two zones, so in main analyses relevant LSOAs assigned to soft water category except for those in local authority districts with any WSZs ≥100 mg/l $CaCO_3$ which remained as missing/excluded.
Thames Water	Yes	Unable to provide data on Ca or Mg for 2012
United Utilities Water	Yes	WSZ-LSOA linkage achieved using postcode sector rather than full postcode
Welsh Water	Yes	Included as some English areas near the Welsh border are supplied by Welsh Water
Wessex Water	Yes	WSZ-LSOA linkage achieved using outer rather than full postcode
Yorkshire Water	Yes	WSZ-LSOA linkage achieved using postcode sector rather than full postcode

Supplementary table 2: ICD-10 code with description
S02, fracture of skull and facial bones
S02.0, fracture of vault of skull
S02.00, fracture of vault of skull
S02.01, fracture of vault of skull
S02.1, fracture of base of skull
S02.10, fracture of base of skull
S02.11, fracture of base of skull
S02.2, fracture of nasal bones
S02.20, fracture of nasal bones
S02.21, fracture of nasal bones
S02.3, fracture of orbital floor
S02.30, fracture of orbital floor
S02.31, fracture of orbital floor
S02.4, fracture of malar and maxillary bones
S02.40, fracture of malar and maxillary bones
S02.41, fracture of malar and maxillary bones
S02.6, fracture of mandible
S02.60, fracture of mandible
S02.61, fracture of mandible
S02.7, multiple fractures involving skull and facial bones
S02.70, multiple fractures involving skull and facial bones
S02.71, multiple fractures involving skull and facial bones
S02.8, fractures of other skull and facial bones
S02.80, fractures of other skull and facial bones
S02.81, fractures of other skull and facial bones
S02.9, "fracture of skull and facial bones, part unspecified"
S02.90, "fracture of skull and facial bones, part unspecified"
S02.91, "fracture of skull and facial bones, part unspecified"
S12, fracture of neck
S12.0, fracture of first cervical vertebra
S12.00, fracture of first cervical vertebra
S12.01, fracture of first cervical vertebra
S12.1, fracture of second cervical vertebra
S12.10, fracture of second cervical vertebra
S12.11, fracture of second cervical vertebra
S12.2, fracture of other specified cervical vertebra
S12.20, fracture of other specified cervical vertebra
S12.21, fracture of other specified cervical vertebra

S12.7, multiple fractures of cervical spine
S12.70, multiple fractures of cervical spine
S12.71, multiple fractures of cervical spine
S12.8, fracture of other parts of neck
S12.80, fracture of other parts of neck
S12.81, fracture of other parts of neck
S12.9, "fracture of neck, part unspecified"
S12.90, "fracture of neck, part unspecified"
S12.91, "fracture of neck, part unspecified"
S22, "fracture of rib(s), sternum and thoracic spine"
S22.0, fracture of thoracic vertebra
S22.00, fracture of thoracic vertebra
S22.01, fracture of thoracic vertebra
S22.1, multiple fractures of thoracic spine
S22.10, multiple fractures of thoracic spine
S22.11, multiple fractures of thoracic spine
S22.2, fracture of sternum
S22.20, fracture of sternum
S22.21, fracture of sternum
S22.3, fracture of rib
S22.30, fracture of rib
S22.31, fracture of rib
S22.4, multiple fractures of ribs
S22.40, multiple fractures of ribs
S22.41, multiple fractures of ribs
S22.5, flail chest
S22.50, flail chest
S22.51, flail chest
S22.8, fracture of other parts of bony thorax
S22.80, fracture of other parts of bony thorax
S22.81, fracture of other parts of bony thorax
S22.9, "fracture of bony thorax, part unspecified"
S22.90, "fracture of bony thorax, part unspecified"
S22.91, "fracture of bony thorax, part unspecified"
S32, fracture of lumbar spine and pelvis
S32.0, fracture of lumbar vertebra
S32.00, fracture of lumbar vertebra
S32.01, fracture of lumbar vertebra
S32.1, fracture of sacrum
S32.10, fracture of sacrum

S32.11, fracture of sacrum
S32.2, fracture of coccyx
S32.20, fracture of coccyx
S32.21, fracture of coccyx
S32.3, fracture of ilium
S32.30, fracture of ilium
S32.31, fracture of ilium
S32.4, fracture of acetabulum
S32.40, fracture of acetabulum
S32.41, fracture of acetabulum
S32.5, fracture of pubis
S32.50, fracture of pubis
S32.51, fracture of pubis
S32.7, multiple fractures of lumbar spine and pelvis
S32.70, multiple fractures of lumbar spine and pelvis
S32.71, multiple fractures of lumbar spine and pelvis
S32.8, fracture of other and unspecified parts of lumbar spine and pelvis
S32.80, fracture of other and unspecified parts of lumbar spine and pelvis
S32.81, fracture of other and unspecified parts of lumbar spine and pelvis
S42, fracture of shoulder and upper arm
S42.0, fracture of clavicle
S42.00, fracture of clavicle
S42.01, fracture of clavicle
S42.1, fracture of scapula
S42.10, fracture of scapula
S42.11, fracture of scapula
S42.2, fracture of upper end of humerus
S42.20, fracture of upper end of humerus
S42.21, fracture of upper end of humerus
S42.3, fracture of shaft of humerus
S42.30, fracture of shaft of humerus
S42.31, fracture of shaft of humerus
S42.4 fracture of lower end of humerus
S42.40, fracture of lower end of humerus
S42.41, fracture of lower end of humerus
S42.7, "multiple fractures of clavicle, scapula and humerus"
S42.70, "multiple fractures of clavicle, scapula and humerus"
S42.71, "multiple fractures of clavicle, scapula and humerus"
S42.8, fracture of other parts of shoulder and upper arm
S42.80, fracture of other parts of shoulder and upper arm

S42.81, fracture of other parts of shoulder and upper arm
S42.9, "fracture of shoulder girdle, part unspecified"
S42.90, "fracture of shoulder girdle, part unspecified"
S42.91, "fracture of shoulder girdle, part unspecified"
S52, fracture of forearm
S52.0, fracture of upper end of ulna
S52.00, fracture of upper end of ulna
S52.01, fracture of upper end of ulna
S52.1, fracture of upper end of radius
S52.10, fracture of upper end of radius
S52.11, fracture of upper end of radius
S52.2, fracture of shaft of ulna
S52.20, fracture of shaft of ulna
S52.21, fracture of shaft of ulna
S52.3, fracture of shaft of radius
S52.30, fracture of shaft of radius
S52.31, fracture of shaft of radius
S52.4, fracture of shafts of both ulna and radius
S52.40, fracture of shafts of both ulna and radius
S52.41, fracture of shafts of both ulna and radius
S52.5, fracture of lower end of radius
S52.50, fracture of lower end of radius
S52.51, fracture of lower end of radius
S52.6, fracture of lower end of both ulna and radius
S52.60, fracture of lower end of both ulna and radius
S52.61, fracture of lower end of both ulna and radius
S52.7, multiple fractures of forearm
S52.70, multiple fractures of forearm
S52.71, multiple fractures of forearm
S52.8, fracture of other parts of forearm
S52.80, fracture of other parts of forearm
S52.81, fracture of other parts of forearm
S52.9, "fracture of forearm, part unspecified"
S52.90, "fracture of forearm, part unspecified"
S52.91, "fracture of forearm, part unspecified"
S62, fracture at wrist and hand level
S62.0, fracture of navicular [scaphoid] bone of hand
S62.00, fracture of navicular [scaphoid] bone of hand
S62.01, fracture of navicular [scaphoid] bone of hand
S62.1, fracture of other carpal bone(s)

S62.10, fracture of other carpal bone(s)

S62.11, fracture of other carpal bone(s)

S62.2, fracture of first metacarpal bone

S62.20, fracture of first metacarpal bone

S62.21, fracture of first metacarpal bone

S62.3, fracture of other metacarpal bone

S62.30, fracture of other metacarpal bone

S62.31, fracture of other metacarpal bone

S62.4, multiple fractures of metacarpal bones

S62.40, multiple fractures of metacarpal bones

S62.41, multiple fractures of metacarpal bones

S62.5,fracture of thumb

S62.50, fracture of thumb

S62.51, fracture of thumb

S62.6, fracture of other finger

S62.60, fracture of other finger

S62.61, fracture of other finger

S62.7, multiple fractures of fingers

S62.70, multiple fractures of fingers

S62.71, multiple fractures of fingers

S62.8, fracture of other and unspecified parts of wrist and hand

S62.80, fracture of other and unspecified parts of wrist and hand

S62.81, fracture of other and unspecified parts of wrist and hand

S72, fracture of femur

S72.0, fracture of neck of femur

S72.00, fracture of neck of femur

S72.01, fracture of neck of femur

S72.1, pertrochanteric fracture

S72.10, pertrochanteric fracture

S72.11, pertrochanteric fracture

S72.2, subtrochanteric fracture

S72.20, subtrochanteric fracture

S72.21, subtrochanteric fracture

S72.3, fracture of shaft of femur

S72.30, fracture of shaft of femur

S72.31, fracture of shaft of femur

S72.4, fracture of lower end of femur

S72.40, fracture of lower end of femur

S72.41, fracture of lower end of femur

S72.7, multiple fractures of femur

S72.70, multiple fractures of femur
S72.71, multiple fractures of femur
S72.8, fractures of other parts of femur
S72.80, fractures of other parts of femur
S72.81, fractures of other parts of femur
S72.9, "fracture of femur, part unspecified"
S72.90, "fracture of femur, part unspecified"
S72.91, "fracture of femur, part unspecified"
S82, "fracture of lower leg, including ankle"
S82.0, fracture of patella
S82.00, fracture of patella
S82.01, fracture of patella
S82.1, fracture of upper end of tibia
S82.10, fracture of upper end of tibia
S82.11, fracture of upper end of tibia
S82.2, fracture of shaft of tibia
S82.20, fracture of shaft of tibia
S82.21, fracture of shaft of tibia
S82.3, fracture of lower end of tibia
S82.30, fracture of lower end of tibia
S82.31, fracture of lower end of tibia
S82.4, fracture of fibula alone
S82.40, fracture of fibula alone
S82.41, fracture of fibula alone
S82.5, fracture of medial malleolus
S82.50, fracture of medial malleolus
S82.51, fracture of medial malleolus
S82.6, fracture of lateral malleolus
S82.60, fracture of lateral malleolus
S82.61, fracture of lateral malleolus
S82.7, multiple fractures of lower leg
S82.70, multiple fractures of lower leg
S82.71, multiple fractures of lower leg
S82.8, fractures of other parts of lower leg
S82.80, fractures of other parts of lower leg
S82.81, fractures of other parts of lower leg
S82.9, "fracture of lower leg, part unspecified"
S82.90, "fracture of lower leg, part unspecified"
S82.91, "fracture of lower leg, part unspecified"
S92, "fracture of foot, except ankle"

S92.0, fracture of calcaneus
S92.00, fracture of calcaneus
S92.01, fracture of calcaneus
S92.1, fracture of talus
S92.10, fracture of talus
S92.11, fracture of talus
S92.2, fracture of other tarsal bone(s)
S92.20, fracture of other tarsal bone(s)
S92.21, fracture of other tarsal bone(s)
S92.3, fracture of metatarsal bone
S92.30, fracture of metatarsal bone
S92.31, fracture of metatarsal bone
S92.4, fracture of great toe
S92.40, fracture of great toe
S92.41, fracture of great toe
S92.5, fracture of other toe
S92.50, fracture of other toe
S92.51, fracture of other toe
S92.7, multiple fractures of foot
S92.70, multiple fractures of foot
S92.71, multiple fractures of foot
S92.9, "fracture of foot, unspecified"
S92.90, "fracture of foot, unspecified"
S92.91, "fracture of foot, unspecified"

Supplementary Table 3: Adjusted incidence rate ratio for fracture hospitalizations acording to total water hardness: by quintiles of deprivation

	Least deprived (Q5)		Less derprived (Q4)		Mid deprived (Q3)		More deprived (Q2)		Most deprived (Q1)	
	Incidence rate ratio	P-value	Incidence rate ratio	P-value	Incidence rate ratio	P-value	Incidence rate ratio	P-value	Incidence rate ratio	P-value
Soft (0-99 mg/l)	ref		ref		ref		ref		ref	
Slightly hard (100-149 mg/l)	0.95 (0.91 - 0.99)	0.015	0.91 (0.87 - 0.95)	<0.001	0.87 (0.84 - 0.91)	<0.001	0.90 (0.86 - 0.94)	<0.001	0.91 (0.88 - 0.94)	<0.001
Moderately hard (149-199 mg/l)	0.93 (0.89 - 0.97)	<0.001	0.89 (0.86 - 0.93)	<0.001	0.88 (0.85 - 0.92)	<0.001	0.87 (0.84 - 0.91)	<0.001	0.97 (0.93 - 1.01)	0.13
Hard (200-299 mg/l)	0.87 (0.84 - 0.89)	<0.001	0.88 (0.86 - 0.91)	<0.001	0.86 (0.84 - 0.88)	<0.001	0.85 (0.83 - 0.87)	<0.001	0.86 (0.84 - 0.89)	<0.001
Very hard (≥300 mg/l)	0.86 (0.83 - 0.89)	<0.001	0.86 (0.83 - 0.89)	<0.001	0.85 (0.83 - 0.88)	<0.001	0.87 (0.84 - 0.90)	<0.001	0.89 (0.85 - 0.92)	<0.001

Supplementary Table 4: Adjusted incidence rate ratios for fracture hospitalisation derived in secondary analyses: overall and by fracture type								
	≥200 mg/l CaCO3 relative to <200 mg/L (reference group)*				CaCO3 analysed on continuous scale with beta coefficients expressed as rate change (fractures per 100,000 person-years) per 100 mg/l increase *			
	Incidence rate ratio	Lower 95% CI	Upper 95% CI	P-value	beta coefficient	Lower 95% CI	Upper 95% CI	P-value
All fractures	0.90	0.89	0.91	<0.001	-16.0	-18.0	-14.0	<0.001
Forearm	0.90	0.89	0.92	<0.001	-6.1	-7.3	-5.0	<0.001
Upper-arm	0.90	0.88	0.92	<0.001	-2.0	-2.6	-1.3	<0.001
Leg	0.93	0.91	0.95	<0.001	-1.4	-2.1	-0.7	<0.001
Skull and face	0.87	0.84	0.90	<0.001	-3.0	-3.5	-2.4	<0.001
Hands and feet	0.88	0.86	0.9	<0.001	-3.4	-4.2	-2.7	<0.001
Other site	0.90	0.85	0.95	<0.001	-0.4	-0.6	-0.1	0.003
Secondary only	0.87	0.83	0.91	<0.001	-0.9	-1.3	-0.6	<0.001

* Lower-layer super output areas included: n=29,776 for binary exposure; n=29,041 for continuous exposure

Supplementary Table 5: Full list of crude and adjusted associations between LSOA socio-demographic factors, total water hardness, and overall fracture incidence in main analysis								
	Boys				Girls			
	Crude		Mutually adjusted		Crude		Mutually adjusted	
	Incidence rate ratio (95% CI)	P-value	Incidence rate ratio (95% CI)	P-value	Incidence rate ratio (95% CI)	P-value	Incidence rate ratio (95% CI)	P-value
**Water hardness**								
Soft (0-99 mg/l)	ref		ref		ref		ref	
Slightly hard (100-149 mg/l)	0.92 (0.90 - 0.94)	<0.001	0.91 (0.89 - 0.93)	<0.001	0.94 (0.92 - 0.97)	<0.001	0.91 (0.87 - 0.94)	<0.001
Moderately hard (149-199 mg/l)	0.91 (0.89 - 0.93)	<0.001	0.91 (0.89 - 0.93)	<0.001	0.93 (0.90 - 0.96)	<0.001	0.91 (0.89 - 0.94)	<0.001
Hard (200-299 mg/l)	0.85 (0.84 - 0.86)	<0.001	0.87 (0.86 - 0.88)	<0.001	0.85 (0.83 - 0.86)	<0.001	0.85 (0.83 - 0.87)	<0.001
Very hard (≥300 mg/l)	0.88 (0.86 - 0.89)	<0.001	0.87 (0.86 - 0.89)	<0.001	0.88 (0.86 - 0.90)	<0.001	0.84 (0.82 - 0.86)	<0.001
**Age proportion (per 10% increase)**								
10-12 years	1.40 (1.16 - 1.64)	<0.001	1.36 (1.16 - 1.61)	<0.001	1.21 (1.17 - 1.25)	<0.001	1.16 (1.13 - 1.20)	<0.001
≥13 years	1.07 (1.06 - 1.08)	<0.001	1.06 (1.05 - 1.07)	<0.001	1.01 (0.99 - 1.02)	0.19	0.97 (0.96 - 0.99)	<0.001
**Deprivation**								
Quintile 5 (Least deprived)	ref		ref		ref		ref	
Quintile 4	1.00 (0.98 - 1.02)	0.83	1.01 (1.00 - 1.02)	0.14	1.00 (0.98 - 1.03)	0.79	1.01 (0.98 - 1.03)	0.63
Quintile 3	1.00 (0.99 - 1.02)	0.66	1.04 (1.02 - 1.05)	<0.001	0.99 (0.97 - 1.01)	0.3	1.00 (0.98 - 1.03)	0.78
Quintile 2	1.00 (0.98 - 1.02)	0.90	1.06 (1.04 - 1.08)	<0.001	0.95 (0.93 - 0.97)	<0.001	0.99 (0.97 - 1.01)	0.26
Quintile 1 (Most deprived)	1.08 (1.06 - 1.09)	<0.001	1.13 (1.11 - 1.15)	<0.001	0.99 (0.97 - 1.01)	0.45	1.02 (1.00 - 1.04)	0.13
**Rurality**								
Rural	ref		ref		ref		ref	
Semi-urban	0.94 (0.93 - 0.96)	<0.001	0.96 (0.95 - 0.98)	<0.001	0.91 (0.89 - 0.92)	<0.001	0.92 (0.90 - 0.94)	<0.001
Urban	0.90 (0.89 - 0.91)	<0.001	0.89 (0.87 - 0.90)	<0.001	0.82 (0.81 - 0.84)	<0.001	0.81 (0.79 - 0.83)	<0.001
**Latitude**								
South	ref		ref		ref		ref	
North	1.09 (1.08 - 1.10)	<0.001	1.01 (1.00 -1.02)	0.12	1.11 (1.09 - 1.12)	<0.001	1.04 (1.02 - 1.06)	<0.001

S1 Fig: Scatter plot of primary care fracture rates by regional water hardness

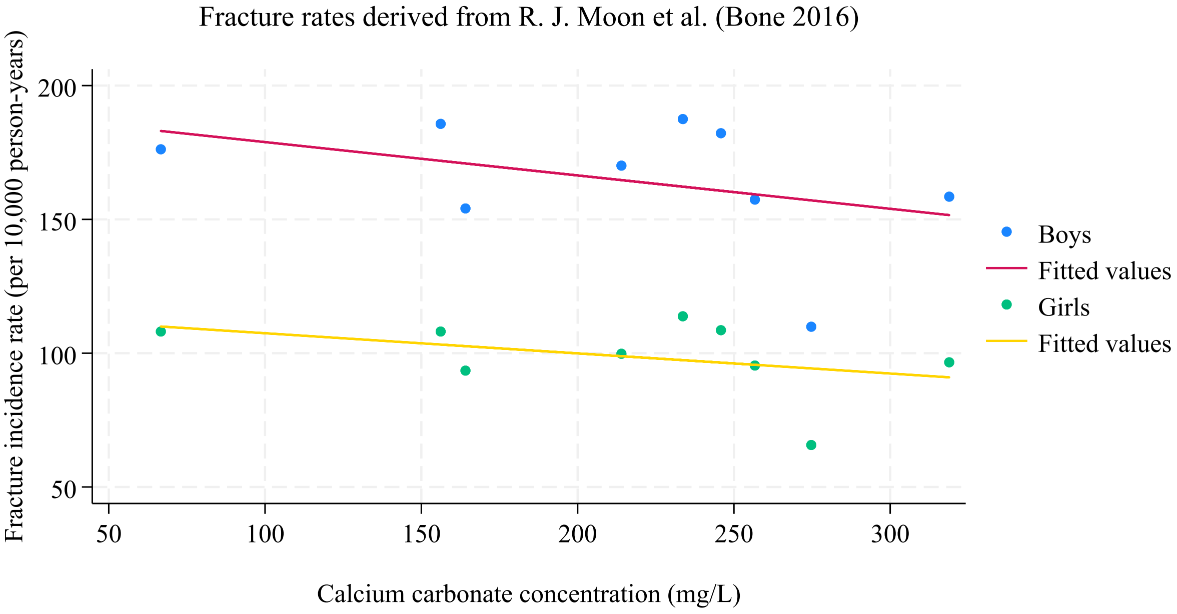

Supplement: Supplementary_Material_ziaf189 [file supplementary_material_ziaf189.pdf]
